# Women with disabilities’ use of maternal care services in sub-Saharan Africa

**DOI:** 10.4102/ajod.v13i0.1327

**Published:** 2024-07-31

**Authors:** Sara H. Rotenberg, Calum Davey, Emily McFadden

**Affiliations:** 1 Nuffield Department of Primary Care Health Sciences, University of Oxford, Oxford, United Kingdom; 2 International Centre for Evidence in Disability, London School of Hygiene and Tropical Medicine, London, United Kingdom

**Keywords:** disability, maternal health, antenatal care, health equity, post-natal care, skilled birth attendance

## Abstract

**Background:**

Quality maternal health care is central to the Sustainable Development Goals efforts to reduce maternal mortality, yet there remain limited quantitative data on maternal care inequities for women with disabilities in sub-Saharan Africa.

**Objectives:**

This study aims to understand the differences in maternal care providers for women with and without disabilities.

**Method:**

We used Multiple Indicator Cluster Surveys from 13 sub-Saharan African countries conducted between 2017–2020. We used logistic and multinomial logistic regression to examine the relationship between disability (Washington Group definition) and antenatal care attendance and the type of care provider for antenatal care, skilled birth attendance, and postnatal and postpartum checks. All analyses were adjusted for age, wealth, country, and location.

**Results:**

The sample included 10 021 women, including 306 (3.1%) women with disabilities. There were small absolute and no relative differences in antenatal care attendance, qualified antenatal care provider, postnatal, and postpartum checks, for disabled and women without disabilities. Women with disabilities had some evidence of higher odds of having a doctor at their birth compared to women without disabilities (aOR = 1.52, 95% CI: 0.99–2.33).

**Conclusion:**

This study shows small absolute and no relative differences between women with and without disabilities for antenatal access and provider types for maternal care, though these findings are limited by a small sample and no data on care quality, acceptability, or outcomes. More research on care quality and outcomes is needed.

**Contribution:**

This study is the first quantitative, multi-country study in sub-Saharan Africa to examine maternal care seeking patterns, demonstrating important data on maternal health indicators for women with disabilities.

## Introduction

Quality maternal healthcare is central to the Sustainable Development Goals (SDGs) efforts to reduce maternal mortality (UN Women 2022). Increasing access to routine antenatal care (ANC), skilled birth attendance, postnatal, and postpartum care can help address high rates of pregnancy-related mortality, particularly in sub-Saharan Africa where maternal mortality remains unacceptably high (Alam et al. 2015; Dahab & Sakellariou 2020; Yaya & Ghose 2019). Critical shortages of health workers have become barriers to expanding maternal health services at sufficient rates (Okoroafor et al. 2022), which has led to a renewed focus on inequities in maternal healthcare across socio-economic (Atake 2021), urban and/or rural (Sidze et al. 2021), and educational (Wang et al. 2021) axes. Despite 12.8% of the sub-Saharan African population experiencing disability in some form there remain limited data on maternal care inequities for women with disabilities in sub-Saharan Africa compared to women without disabilities (World Health Organization [WHO] 2022).

Women with disabilities face similar barriers in seeking maternal care as they do for healthcare more broadly, including health workers’ poor attitudes, limited access to transportation to travel for care, and a lack of inclusive health information (Ganle et al. 2016; Heideveld-Gerritsen et al. 2021; Kuper & Heydt 2019). There is some evidence that women with disabilities have higher rates of mortality, complications, and worse quality care in high-income and some sub-Saharan African settings (Apolot et al. 2019; Ayiasi et al. 2013; Brown et al. 2022; Ganle et al. 2016; Hameed & Asim 2020; Heideveld-Gerritsen et al. 2021; Malouf, Henderson & Redshaw 2017a, Malouf et al. 2017b, Mitra et al. 2017a, 2015; Tarasoff et al. 2020). However, quantitative research on maternal care for women with disabilities is severely lacking in sub-Saharan Africa. The inclusion of the Washington Group Short Set (WG-SS) in Round 6 of the United Nations International Children’s Emergency Fund (UNICEF)-supported Multiple Indicator Cluster Surveys (MICS) provides an opportunity to investigate inequities in key maternal care indicators and access to trained health workers for women with disabilities compared to women without disabilities (Hancioglu & Arnold 2013; Khan & Hancioglu 2019; UNICEF 2017). Accordingly, we analysed data from 13 countries in sub-Saharan Africa that had completed the MICS6 survey to compare women with disabilities’ care seeking patterns for maternal care to women without disabilities. We examined patterns in seeking ANC and from whom they sought antenatal care, skilled birth attendance, and postnatal and postpartum checks.

## Methods

### Data source

The MICS are the largest set of internationally comparable household datasets used to measure the health and development status of women and children in low- and middle-income countries (Khan & Hancioglu 2019). Data were from the sixth round of the MICS, conducted between 2017 and 2020 in 13 sub-Saharan African countries (Central African Republic, Chad, Democratic Republic of Congo, Gambia, Ghana, Guinea-Bissau, Lesotho, Madagascar, Malawi, São Tomé and Príncipe, Sierra Leone, Togo, and Zimbabwe). Analysing each individual dataset was not possible because of small sample sizes, so a pooled sample of all countries in the region was used.

### Disability measures

The MICS6 surveys have an adult functioning module, which uses the WG-SS to assess functional impairment in individual men and women aged 18–49 (Cappa et al. 2018; Loeb et al. 2018). This takes about 10 min to administer and measures impairment in six functional domains: seeing, hearing, walking or climbing steps, remembering or concentrating, self-care, and communication (Loeb et al. 2018; Zia et al. 2020).

In this study, we utilised the Washington Group on Disability Statistics Guidelines thresholds for disability. Women were coded as disabled if they reported functional difficulty in any of the domains (i.e., ‘cannot do at all’ or ‘a lot of difficulty’ to any of the questions). Women who reported functional difficulty in at least one domain, but had other domains missing were coded as disabled as the missing sections do not impact their disability status for the purposes of this study (The Washington Group on Disability Statistics n.d.). Women who responded ‘no difficulty’ or ‘some difficulty’ to all functional domains were coded as women without disabilities. Individuals with missing data on sex, who had not given birth in the last 2 years, or had missing data for all WG-SS questions were excluded, as their birth history and disability status could not be determined (The Washington Group on Disability Statistics n.d.). A sensitivity analysis was conducted and our treatment of missing data were not found to impact the results.

### Outcomes

This study measured maternal care for a woman’s most recent birth and had four main outcomes: antenatal care coverage, skilled birth attendance provider, post-natal care provider, and postpartum care provider. Antenatal care attendance was measured by whether or not a woman saw someone (trained or untrained) for antenatal care at least once during a pregnancy in the last 2 years. Variables measuring the provider type for antenatal care, skilled birth attendance, were grouped into nurses/midwives, physicians, community carers (traditional birth attendant, community health worker), and untrained individuals or no one (relative or friend, no one) based on WHO’s International Standard Classifications of Occupations (ISCO-08) (International Labour Organisation 2011) compared to the MICS survey response options. These groupings were selected based on the WHO guidelines for maternal healthcare that encourage task-shifting from physicians to trained health workers for maternal care, such as nurses/midwives (ed. WHO 2017). Community health workers were included in the community carers second group as the WHO guidelines recommend their participation in providing maternal care, but prefer skilled birth attendance from a physician, nurse, or midwife for comprehensive care (ed. WHO 2017).

### Statistical analysis

Analyses were completed using R version 4.2.2. We calculated baseline summary statistics (means and standard deviations or numbers and proportions) for all outcomes and covariates for the pooled sample of all 13 countries (Central African Republic, Chad, Democratic Republic of Congo, Gambia, Ghana, Guinea-Bissau, Lesotho, Madagascar, Malawi, Sao Tome et Principe, Sierra Leone, Togo, Zimbabwe) overall and by disability status.

We used logistic regression to understand the relationship between disability status and antenatal care attendance for the region overall. This was adjusted for age, wealth, country, and location (urban and/or rural). Multinomial logistic regression was used to examine the relationship between disability status and the type of health worker who provided antenatal care, skilled birth attendance, and post-natal checks. Analyses are as a combined estimate for all countries, impairment types, and levels of impairment because of small sample sizes. All analyses were adjusted for age, wealth, country, and location (urban and/or rural). All models accounted for the clustered survey design (i.e., country, cluster, household numbers, and sample weights) and used robust standard errors for the confidence interval calculations.

### Ethical considerations

Anonymised data were obtained from UNICEF from their website where all MICS data are publicly available (http://www.mics.unicef.org/). Ethical clearance and informed consent was the responsibility of the national statistical institutions or UNICEF-partner institutions who administered the survey. This is an analysis of secondary data and patients, or the public, were not involved in the study design or analysis. As we only had access to publicly available, anonymised data this study was exempt from the University of Oxford ethics review.

## Results

This study included 10 021 women between the ages of 18 and 49 years, who had reported a live birth in the past 2 years, from 13 countries in sub-Saharan Africa. Baseline characteristics are shown overall and by disability status in Table 1. The mean age was 27.8 ± 6.4 years in women without disabilities and 28.7 ± 6.9 years in women with disabilities. Fewer women with disabilities lived in urban areas than women without disabilities (28.4% vs. 31.7%). The proportion of women with disabilities in the lowest wealth category was higher than in women without disabilities (37.9% vs. 30.4%). Overall, the prevalence of disability was 3.1% (*n* = 306). The proportion of women receiving at least one ANC visit from any provider type was high at 95.4% (*n* = 9030) and there were small absolute differences between disabled and women without disabilities (91.8% vs. 95.5%). Both women with and without disabilities saw nurses the most for their maternal care compared to other health worker cadres (qualified ANC: 84.2% vs. 86.9%; skilled birth attendance: 60.4% vs 67.0%; postnatal check: 81.2% vs. 83.7%; and postpartum check: 78.9% vs. 82.1%), although there were small absolute differences.

**TABLE 1:** Baseline characteristics, overall and by disability status, of 10 021 women from 13 Multiple Indicator Cluster Surveys in sub-Saharan Africa, 2017–2020.

Variable	Overall		No disability		Disability	
	*n*	%	*n*	%	*n*	%
Total	10 021	100.0	9715	96.9	306	3.1
Age	27.8	6.5	27.8	6.4	28.7	6.9
Urban	3171	31.6	3084	31.7	87	28.4
Disability prevalence	306	3.1	-	-	-	-
**Wealth quintiles**						
Bottom	3069	30.6	2953	30.4	116	37.9
2	2245	22.4	2184	22.5	61	19.9
3	1879	18.8	1821	18.7	58	19.0
4	1602	16.0	1559	16.1	43	14.1
Top	1224	12.2	1196	12.3	28	9.2
**Antenatal care attendance**						
At least one visit	9030	95.4	8773	95.5	257	91.8
**Qualified ANC provider**						
Nurse or Midwife	7573	86.9	7370	86.9	203	84.2
Doctor	1106	12.7	1069	12.6	37	15.4
Community Health Provider	40	0.5	39	0.5	1	0.4
**Skilled birth attendance**						
Nurse or Midwife	6265	66.8	6099	67.0	166	60.4
Doctor	1131	12.1	1088	11.9	43	15.6
Community Health Provider	1195	12.7	1155	12.7	40	14.5
No one/friend	789	8.4	763	8.4	26	9.5
**Postnatal check on baby**						
Nurse or Midwife	3961	83.7	3853	83.7	108	81.2
Doctor	339	7.2	330	7.2	9	6.8
Community Health Provider	301	6.4	290	6.3	11	8.3
Friend/relative	133	2.8	128	2.8	5	3.8
**Postpartum check on mother**						
Nurse or Midwife	3034	82.0	2948	82.1	86	78.9
Doctor	244	6.6	238	6.6	6	5.5
Community Health Provider	243	6.6	230	6.4	13	11.9
Friend or relative	180	4.9	176	4.9	4	3.7

ANC, antenatal care.

### Antenatal care

Antenatal care coverage and provider types are shown in Table 2. There was no strong evidence that women with disabilities had different ANC attendance compared to women without disabilities (adjusted OR = 0.64, 95% C.I. 0.39–1.05) or that women with disabilities saw different types of ANC providers compared to women without disabilities (doctors: aOR = 1.25, 95% C.I. 0.81–1.91; community health providers: aOR = 0.51, 95% C.I. 0.06–4.28).

**TABLE 2:** Adjusted odds ratios for antenatal care attendance and providers types for women with disabilities compared to women without disabilities in 13 Multiple Indicator Cluster Survey countries in sub-Saharan Africa, 2017–2020.

Provider categories	Disability (*N*)	Country-adjusted only for women with disabilities		Adjusted† for women with disabilities	
		OR	95% C.I.	OR	95% C.I.
**Antenatal care attendance**					
At least one visit	257	0.64	0.39, 1.05	0.64	0.39, 1.05
**Antenatal care provider type**					
Nurse or midwife	203	1.00	-	1.00	-
Doctors	37	1.23	0.82, 1.88	1.25	0.81, 1.91
Community Health Provider	1	0.57	0.07, 4.71	0.51	0.06, 4.28

† , Adjusted for country, wealth, location, and age.

Table 3 shows adjusted odds ratios for which a cadre of health worker (nurses or midwives, doctors, community health providers, and a friend or no one) provided care for each of the three outcomes: skilled birth attendance, postnatal and postpartum care.

**TABLE 3:** Adjusted odds ratios for the care provider type for maternal healthcare for women with disabilities compared to women without disabilities from 13 Multiple Indicator Cluster Survey countries in sub-Saharan Africa, 2017–2020.

Indicator	Nurse or midwife		Doctors			Community health provider			No one or friend/relative		
	*n*	ref.	*n*	OR	95% C.I.	*n*	OR	95% C.I.	*n*	OR	95% C.I.
**Country-adjusted only OR [95% C.I.] for women with disabilities**											
Skilled birth attendance	166	1.00	43	1.40†	0.93, 2.11	40	0.86	0.51, 1.45	26	0.90	0.53, 1.53
Postnatal health checks on baby	108	1.00	9	0.99	0.44, 2.20	11	0.88	0.32, 2.46	5	0.80	0.19, 3.26
Postpartum health checks on mother	86	1.00	6	0.82	0.29, 2.34	13	1.58	0.57, 4.35	4	0.35	0.84, 1.48
Adjusted* OR [95% C.I.] for women with disabilities											
Skilled birth attendance	166	1.00	43	1.52	0.99, 2.33	40	0.70	0.41, 1.18	26	0.72	0.42, 1.25
Postnatal health checks on baby	108	1.00	9	0.89	0.40, 1.99	11	0.77	0.30, 2.00	5	0.79	0.19, 3.26
Postpartum health checks on mother	86	1.00	6	0.95	0.34, 2.67	13	1.38	0.51, 3.75	4	0.35	0.87, 1.38

* , Adjusted for country, wealth, location, and age.

† , Each care provider category is compared to nurses or midwives for women with disabilities compared to women without disabilities. In practice, this is interpreted as ‘women with disabilities had between 0.93 times lower odds and 2.11 times higher odds of seeing doctors compared to nurses or midwives compared to women without disabilities’.

### Skilled birth attendance

There was no strong evidence that women with disabilities had different birth attendants than women without disabilities (Table 3: doctors: aOR = 1.52, 95% C.I. 0.99–2.33; health workers: aOR = 0.70, 95% C.I. 0.41–0.1.18; no one or a friend: aOR = 0.72, 95% C.I. 0.42–1.25). However, there was some evidence that women with disabilities more often had their births attended to by doctors.

### Postnatal care

There was no evidence of a difference in care provider for women with and without disabilities for the postnatal checks on their babies (Table 3: doctors [aOR = 0.89, 95% C.I. 0.40–1.99], community health providers [aOR = 0.77, 95% C.I. 0.30–2.00], no one or friend/relative [aOR = 0.79, 95% C.I. 0.19–3.26]).

### Postpartum care

Care provider types for postpartum care for women with and without disabilities also showed no differences between the groups (Table 3: doctors [aOR = 0.95, 95% C.I. 0.34–2.67], community health providers [aOR = 1.38, 95% C.I. 0.51–3.75], no one or friend/relative [aOR = 0.46, 95% C.I. 0.87–1.38]).

## Discussion

This study examined the differences in care seeking patterns and care providers for women with and without disabilities. In the sample of 10 021 women, estimates for antenatal care attendance, qualified antenatal care provider, postnatal, and postpartum checks, showed small absolute and no relative differences between women with and without disabilities, although these estimates were imprecise, with wide confidence intervals. There was some evidence that women with disabilities were attended to by doctors more often than women without disabilities (aOR: 1.52, 95% C.I. 0.99–2.33) during birth.

Underpinning these findings is the general context of inequities in maternal healthcare in sub-Saharan Africa. For example, several studies have highlighted the rural–urban and wealth divide that results in worse access to maternal health services for rural and poorer women, respectively (Alam et al. 2015; Samuel, Zewotir & North 2021). While we controlled for these factors, our analysis focusing on disability-based inequities still highlights another dimension of inequities within maternal health in sub-Saharan Africa. Our findings in this analysis largely diverge from other literature that highlights these substantial inequities for women with disabilities. For example, evidence from a systematic review (Heideveld-Gerritsen et al. 2021) and studies in Ghana (Ganle et al. 2016) and Uganda (Apolot et al. 2019) suggested that these barriers include communication, support, transportation, accessible health facilities, basic needs during delivery, and stigma or discrimination from health workers throughout the birth process. However, these barriers do not seem to translate into antenatal care differences according to our results compared with other household survey literature. For instance, evidence from Pakistan’s Demographic and Health Survey did not show inequities in use of antenatal care between women with and without disabilities (Hameed & Asim 2020) and UK research that has shown that women with disabilities have comparable access to antenatal care, in line with our study. This previous literature and our findings suggest that there is sufficient access to antenatal care compared to the general population, although these studies have not explored the possible barriers in the affordability and quality of care.

No studies have examined the different types of care provider women with disabilities see for their maternal care, making it difficult to compare with existing literature. However, our findings did not show differences in care providers for postnatal and postpartum care. There was some evidence that there was a small difference in skilled birth attendance, but this was not statistically significant. Importantly, there was no measure of quality or acceptability of care within these indicators, which is a common issue for women with disabilities in the literature. For instance, national surveys and qualitative studies, showed that women with disabilities in the UK lack the support to make maternal care and childbirth a safe and supportive experience (Malouf et al. 2017a, 2017b). More research is needed to understand this important element of maternal care for women with disabilities.

Therefore, this study highlights the fact that the relationship between women with disabilities and the health system is a complex balance between medical need, rights, and preferences. This complexity requires that maternal health outcomes are evaluated in context. For example, our results suggest women with disabilities may have higher odds of skilled birth attendance from doctors (aOR = 1.52, 95% C.I. 0.99 to 2.33) than women without disabilities. Having a doctor as a skilled birth attendant may indicate a health centre or hospital birth, which may be appropriate as previous research has suggested higher risks of complications for both the mother and the baby (Brown et al. 2022; Mitra et al. 2015; Tarasoff et al. 2020). This finding may contribute to the fact that women with disabilities have more facility-based births, but this should not necessarily infer better quality care necessarily.

A mix of qualitative and quantitative studies has found that women with disabilities feel they are less likely to be adequately supported during birth or by healthcare professionals than women without disabilities (Devkota et al. 2017; Ganle et al. 2016; Malouf et al. 2017a; Redshaw et al. 2013). These factors, along with the other social factors such as poverty and poor education make women with disabilities more susceptible to adverse outcomes (Atake 2021). As women with disabilities usually have lower levels of health insurance, income, and are more likely to be in poverty, the facility-based birth may be a catastrophic health expenditure, pushing women with disabilities further into poverty (Mitra et al. 2017b). Moreover, facility-based births might not be in-line with a woman’s desired birth plans and the data do not allow us to explore whether the nature of a woman’s impairment led to the choice for a facility-based birth. Qualitative studies around the world have also shown that women with physical impairments report their maternal care is not often aligned with their desires or adaptable and responsive to their wishes (Ganle et al. 2016; Heideveld-Gerritsen et al. 2021; Tarasoff 2017). Given autonomy and quality of care were not examined in this study, our findings should not necessarily be inferred as better care, but rather examined more fully in further research on maternal outcomes of women with disabilities.

While the differences in care providers for other forms of maternal care were inconclusive, the small sample size highlights the need to expand disability disaggregation in other maternal health surveys and across intersectional factors (i.e., race, socio-economic status, religion, etc.). The lack of inclusion of disability status in core SDG indicators, including maternal healthcare, impacts our ability to understand inequities for women with disabilities, although we broadly understand that people with disabilities are not yet ‘expected, accepted, or connected’ within the health system, as per the Missing Billion Health System Framework (The Missing Billion Initiative and Clinton Health Access Initative 2022).

### Strengths and limitations

This study provides new evidence on maternal healthcare for women with disabilities in sub-Saharan Africa, using data from a large, nationally representative household survey. While other studies have used Demographic and Health Surveys (Hameed & Asim 2020) or have been conducted in other countries (Brown et al. 2022; Malouf et al 2017a, Mitra et al. 2017a, 2015; Tarasoff, 2017), this study provides data on critical SDG indicators across multiple countries in sub-Saharan Africa. Furthermore, specifically examining which types of health workers women with disabilities reported seeking care from provides important data on which care may be most accessible and where further efforts to improve access and quality could be focused to reach women with disabilities.

Our study was limited by the small proportion of women with disabilities compared to other estimates of disability prevalence (World Bank and World Health Organization 2011), which translated to imprecise estimates and an inability to stratify results by country, impairment type, and level of impairment. While this study did control for country in the analysis, the lack of country disaggregation potentially masks important country-specific trends or system-level factors, such as health insurance coverage, transport subsidies, and outreach programmes that may impact country-level results. However, even at a regional level, there was a small sample size and the proportion of women with disabilities in this study substantially differs from previous estimates (3% in this study vs. 15.4% in the literature), (World Bank & World Health Organization 2011) including other age groups in the MICS surveys (Rotenberg, S, Kuper, H, Davey, C, unpublished data). This is likely because the Washington Group Short Set has several notable limitations, including a narrower definition of functioning and functional domains than the MICS Child Functioning Module and WHO Model Disability Surveys and the fact that the domains in the short set are less sensitive to including people with psychosocial, intellectual, or developmental impairments. This is an important omission, as these groups face particular barriers in accessing sexual and reproductive healthcare. Moreover, as this is self-reported data on disability and there are no published details on accessibility considerations in the survey design (i.e., interpreters, accessible formats, etc.), the sample may be biased by non-response, narrow definitions of disability, and only include individuals with lower thresholds of impairment who can participate in the survey without accommodations. This is an important limitation, particularly in relation to the findings that suggest fewer than expected differences for women with disabilities.

Additionally, the survey bases questions on sex, rather than gender identity, and therefore may not be fully inclusive of people who have given birth in the past two years, although this is unlikely to impact our results. Finally, the data provide no indication on the quality of care women with disabilities received after seeking care, including how their wishes around birth are respected, which is a consistent issue in the literature on health care for people with disabilities. Further research is needed to understand these trends in the context of high-quality care and respect for women’s preferences.

## Conclusion

In summary, our results show no evidence of differences between women with and without disabilities for antenatal attendance, antenatal care provider, and postnatal and postpartum check-up providers. It provides some evidence that women with disabilities have higher odds of having doctors as skilled birth attendants, but this association was not seen at the *p* < 0.05 significance level. Our study was limited by a smaller than expected proportion of women with disabilities and no data on the quality of care or birth outcomes. Thus, there is a need for further studies in sub-Saharan Africa to examine whether interventions to reduce maternal mortality, improve maternal care, and ensure the maternal-health focused SDG efforts are reaching women with disabilities. Improving the accessibility and quality of care for women with disabilities is particularly important as we destigmatise pregnancy and parenthood among people with disabilities and countries increasingly deliver on people with disabilities’ rights to sexual and reproductive health. Without better data on the maternal care seeking patterns and experiences, the lack of data masks inequity for people with disabilities. To deliver high-quality health systems, we need to expand research into maternal health outcomes and quality improvement for women with disabilities.
